# Sir Andrew Clark Taken to Task

**Published:** 1889-06-29

**Authors:** 


### The Hospital

A Weekly Institutional Journal of

Science, Medicine, Nursing, and Philanthropy.

*For Hospitals, Asylums, Medical Practitioners, Students, Nurses, Families, Parishes, Congregations, and the Charitable Public.*

Vol. VI. No. 144.] [June 29, 1889.

### Sir Andrew Clark Taken to Task.

A great opportunity was given to Sir Andrew Clark when he was asked to deliver what may be considered the oration on behalf of hospitals at the Mansion House on Wednesday week. As the delivery of such an oration will probably become annual, and as, moreover, its effect upon hospitals, and the general estimation in which they are held by the public are likely to be much influenced by it, a little consideration devoted to the performance will not be waste of time. Sir Andrew Clark was at his best before a popular audience, and spoke eloquently and well: but certain parts of his speech call for a word of protest.

Sir Andrew's first object seems to have been to strike terror into the souls of his audience. Civilisation has many triumphs to display, but what of its failures? asked the orator. In front of the Mansion House, where he was speaking, those failures might, he said, be seen in large numbers. Anxious faces, sad faces, sickly faces were there on all hands. Then Sir Andrew advanced this startling proposition, which if true demands more thought than is implied by a passing cheer at a public meeting. He said, "The advance of civilisation is making more and more difficult the possibilities of life and health." Is that really so? Is the death-rate increasing? Are epidemics more prevalent? Do smallpox, scarlet fever, diphtheria, and typhoid fever attack the people in larger numbers and destroy them in increasing proportion? Are the lives of the rich and poor shorter than they were? If that be true, then the opposite statements, which we hear so often from eminent medical men, must be the opposite of true—must, in short, be false. For we are told every day of the triumphs of medicine, not only in alleviating pain, but also in increasing the general longevity. Moreover, insurance offices seem to believe that the health and life prospects of the community have decidedly improved, and they have in consequence gone in for a general lowering of their rates. Yet here we have the President of the College of Physicians telling us in the most serious and monitory tones that "the advance of civilisation is making more and more difficult the possibilities of life and health." Not only so, but "our capacity for readjusting our sanitary knowledge and applying it to the business of life are, for the present at least, pretty nearly at an end." Nay worse, for civilisation will still advance, according to Sir Andrew, whether our capacities for readjusting and practically applying our sanitary knowledge are at an end or not. "A civilisation still advancing will beget new accidents and new diseases, and will render health more and more difficult to preserve in the midst of such a great city as this." Is this to be taken seriously, or was it merely a display of platform rhetoric, intended to serve a momentary purpose, and destined to die with the occasion? If it expressed the sober and final conclusions of one of the most eminent and scientific physicians of the times, then, indeed, England and London are in a bad way. Civilisation is a failure, medical science is a failure, the hospitals are failures, Sir Andrew Clark and his eminent brethren are failures, and pessimism—stark, staring, detestable pessimism—is victoriously triumphant. We cannot believe that this is Sir Andrew Clark's final and deliberate judgment. He surely stated too strongly the real point he wished to make! Or perhaps he was incorrectly reported! If he was not either overstating his case or badly reported, but uttering his deliberate convictions, then he established the strongest possible case, not for, but against civilisation, against medical science, and against the very hospitals themselves, on behalf of which he was pleading.

The orator made a decided point when he sketched in a few rapid sentences the rise and progress of an imaginary Hospital for "Diseases of the Great Toe." True, the element of originality was wanting. A history of the "Great Toe Hospital" appeared in these pages long ago, and no doubt most of Sir Andrew's audience were familiar with it. But it will bear repetition; and, at any rate, the disgraceful increase of unnecessary special hospitals cannot be too strongly reprehended or too strenuously resisted. It may, however, be doubted whether the rehashing of such a stale story was quite compatible with the dignity either of Sir Andrew Clark in his private capacity or of the president of the Royal College of Physicians. The occasion, it seems to us, was one not so much for *ad captandum* flights of rhetoric as for serious and earnest reasoning on the higher levels of economic value and moral duty.

What could have possessed Sir Andrew Clark to make such an indiscriminate onslaught on rate-supported hospitals? Of course, it is easy to see that he wanted to show how very white the voluntary hospitals were, and he thought the best way to do this was to paint the rate-supported hospitals black. But, although we give place to none in our allegiance to the voluntary principle as against rate-support, we have to ask, in the name of justice and fairness, whether it is certainly true that the rate-supported hospital is necessarily and always "cold, lifeless, mechanical, devoid of the feeling of personal interest and love," . . . . "starving the moral faculties of that exercise which gives them their life," and nobody knows what? Is it really the case that its work is "sorry work," and that there is not one such institution which could be set up as an example of what a hospital ought to be? Let it be remembered that, notwithstanding certain objectionable features, these rate-supported hospitals are manned by responsible members of the medical profession; that excellent nurses and probationers are in charge of the patients; and that the hospital buildings are in some cases the newest, best constructed, and healthiest in the kingdom. How does Sir Andrew Clark justify such strictures on a whole class of men and women, many of whom are as moral, as kind, and as devoted to their work as he is himself?

The out-patient department, just as it is, with all its proved abuses on its head, has a truculent defender in Sir Andrew Clark. What the general practitioners who meet Sir Andrew in consultation will think of his attitude it would be highly interesting to know. But the orator proved too much. He admitted that 8 per cent. of the out-patients at Manchester could have contributed something to a provident dispensary. But why mention one hospital alone? Why did he not take the statements of men who know as much, and probably a great deal more, about the financial circumstances of out-patients than he does, and let his audience know there were two sides to the question? There are those who have studied the subject thoroughly, who assert that not 8 but 40 per cent. of out-patients at some hospitals could afford to pay doctor's fees. The out-patient question is not exhausted when it is shown that at one hospital, or in one city, there is not more than one in every ten or twelve out-patients who could pay for a doctor. It is surely worth a consideration that there are hundreds, probably thousands, of general practitioners whose business is injured by the competition of hospitals, many of whom are in much greater financial straits than some of the out-patients. The present writer knew intimately a man who sent his child for weeks to the out-patient department of a hospital, who had at the very moment of doing so an income of at least £1,400 a year, and who, when he died two years later, left realised property to the amount of £20,000. Undoubtedly cases almost as bad as this are numerous. Whatever Sir Andrew Clark may say, there is not the slightest question that in London the out-patient department at many hospitals is abused to a degree which is shameful in itself, injurious to outside medical practice, and a source of great moral depravation to large numbers of the people.

It seems ungracious to add more to these already sufficiently numerous strictures; but we wish to point out that Sir Andrew Clark failed as much in his duty in one particular as he exceeded it in several others. He proclaimed the services which one newspaper was rendering to the Hospital Sunday Fund, but carefully refrained from so much as mentioning any other paper, although all the leading dailies and weeklies have from year to year written leading articles and opened their columns in every possible way in furtherance of the fund's interests. Why this boycotting of every newspaper in the metropolis in order that the greater glory may be given to one? The Hospital Sunday Fund is a popular institution, and can only survive and prosper by means of popular aid. If every newspaper boycotted this year by Sir Andrew Clark in his capacity of orator were to turn the tables next year by boycotting the Sunday Fund, the managers would hardly thank their orator for the very doubtful services he had rendered to their institution.

